# Effect of anaerobic or/and microaerophilic atmosphere on microcosm biofilm formation and tooth demineralization

**DOI:** 10.1590/1678-7757-2022-0445

**Published:** 2023-06-02

**Authors:** Aline Silva BRAGA, KIM Rafaela Ricci, Ana Carolina MAGALHÃES

**Affiliations:** 1 Universidade de São Paulo Faculdade de Odontologia de Bauru Departamento de Ciências Biológicas Bauru SP Brasil Universidade de São Paulo, Faculdade de Odontologia de Bauru, Departamento de Ciências Biológicas, Bauru, SP, Brasil.

**Keywords:** Biofilms, Dental caries, Dental enamel, Dentin, Microorganisms

## Abstract

Microcosm biofilms can reproduce the complexity of a dental biofilm. However, different forms of cultivation have been used. The impact of the culture atmosphere on the development of microcosm biofilms and their potential to cause tooth demineralization has not yet been deeply studied.

**Objective:**

This study analyzes the effects of three experimental cultivation models (microaerophile vs. anaerobiosis vs. experimental mixed) on the colony-forming units (CFU) of the cariogenic microorganisms and tooth demineralization.

**Methodology:**

90 bovine enamel and 90 dentin specimens were distributed into different atmospheres: 1) microaerophilia (5 days, 5% CO_2_); 2) anaerobiosis (5 days, jar); 3) mixed (2 days microaerophilia and 3 days anaerobiosis), which were treated with 0.12% chlorhexidine (positive control – CHX) or Phosphate-Buffered Saline (negative control – PBS) (n=15). Human saliva and McBain’s saliva containing 0.2% sucrose were used for microcosm biofilm formation, for 5 days. From the second day to the end of the experiment, the specimens were treated with CHX or PBS (1x1 min/day). Colony-forming units (CFU) were counted, and tooth demineralization was analyzed using transverse microradiography (TMR). Data were subjected to two-way ANOVA and Tukey’s or Sidak’s test (p<0.05).

**Results:**

CHX was able to reduce total microorganism’s CFU compared to PBS (differences of 0.3–1.48 log_10_ CFU/mL), except for anaerobiosis and microaerophilia in enamel and dentin biofilm, respectively. In the case of dentin, no effect of CHX on Lactobacillus spp. was observed. CHX significantly reduced enamel demineralization compared to PBS (78% and 22% reductions for enamel and dentin, respectively). Enamel mineral loss did not differ when compared with the other atmospheres; however, the enamel lesion depth was greater under anaerobiosis. Dentin mineral loss was lower under anaerobiosis when compared with the other atmospheres.

**Conclusion:**

The type of atmosphere has, in general, little influence on the cariogenic ability of the microcosm biofilm.

## Introduction

Dental caries is a multifactorial disease of great clinical relevance, which occurs due to the presence of a biofilm in dysbiosis, induced by the frequent ingestion of sugar, which is rich in acidogenic, aciduric, and extracellular polysaccharide-producing microorganisms. These microorganisms can metabolize different types of sugars from the diet, but mainly sucrose, producing acids that change the biofilm pH and cause tooth demineralization.^1-3^ The most common microorganisms involved in the development of dental caries are *Streptococcus mutans* and lactobacilli.^4,5^

Due to the high degree of controlled conditions and reproducibility, *in vitro* models have been well accepted to mimic cariogenic biofilms.^6^ *In vitro* models can be produced from monospecies (namely, *S. mutans*) or multispecies (namely, *S. mutans* + *Lactobacillus casei*) biofilms by using microbial strains or from microorganisms derived from human saliva or dental biofilms, called microcosm biofilm.^7,8^

Microcosm biofilms are able to reproduce the complexity of a dental biofilm.^9-11^ However, different forms of cultivation have been used comprising 24-well microtiter plates, where specimens can be attached to the bottom of the wells;^12,13^ the application of meshes for the retention of microorganisms;^14^ or the suspension of specimens to prevent the formation of biofilm only by microorganism precipitation.^15^ Some microcosm biofilm models use artificial mouths for programmed or continuous nutrient flow.^6,16^

Studies also differ with respect to the sucrose concentrations and form of exposure, which can be either continuous or intermittent.^17,18^ Cultivation time can vary from 2–14 days depending on the selected response variables.^13,19^ Incubation atmosphere is another important factor,^13^ which can vary between microaerophilia (5%–10% CO_2_);^12,14,16,19^ and anaerobiosis with the use of jars, candles, or anaerobic cabins (O_2_<0.1% and 5%–10% CO_2_; 10% CO_2_, 10% H_2_, and 80% N_2_).^17,20^

Oral dental biofilms are often subjected to fluxes of environmental conditions, such as fast pH challenges, nutrient abundance or scarcity, different carbohydrates exposures, and variations in redox potentials due to atmospheric conditions. These environmental parameters can define the biofilms microbiome^21,22^ and, consequently, the cariogenic potential of biofilm.^10,23^

Despite this wide methodological variation between studies, the impact of the culture atmosphere on the development of microcosm biofilms and their potential to cause tooth demineralization has not yet been deeply studied. Therefore, this study aims to compare the three experimental cultivation models (microaerophile vs. anaerobiosis vs. experimental mixed) on the colony forming units (CFU) of the cariogenic microorganisms and tooth demineralization. The null hypothesis is that the type of atmospheric do not influence the *S. mutans* and *Lactobacillus* spp. colonies and development of dental caries lesions (tooth demineralization).

## Methodology

### Saliva Collection

This study was approved by the local ethics committee (CAAE: 35403320.1.0000.5417). The study was conducted according to the Declaration of Helsinki. After the signed consent, ten healthy participants (age: 23.8 ± 3 years old; eight women and two men) donated stimulated saliva for 10 min during morning time, according to the inclusion criteria: (1) normal salivary flow (stimulated saliva flow > 1 mL min and non-stimulated saliva flow >0.3 mL min), (2) with a previous history of caries but no active caries, (3) without gingivitis/periodontitis, and (4) without the ingestion of antibiotics for three months prior to the experiment. Prior to the day of collection, the donors did not brush their teeth. Furthermore, they were not allowed to ingest food or drinks within the last 2 h before saliva collection.^24^ Saliva pool was diluted in glycerol (70% saliva and 30% glycerol), and aliquots of 1 mL were stored at −80 °C.^25^

### Tooth specimen preparation

Bovine teeth were obtained from food manufacturing industry (Frigol S. A., Lençóis Paulista, São Paulo, SP, Brazil). The study was approved by the Ethics Committee on Animal Research (CEUA, Number 005/2020) following the guidelines provided by the National Council for Control of Animal Experimentation. In total, 90 bovine enamel and 90 bovine root dentin specimens (4 mm × 4 mm) were polished and evaluated regarding the average roughness (Ra; contact profilometer Mahr, Göttingen, Germany)^24^ to standardize the tooth surfaces for biofilm growth. The specimens’ surface was divided in three-thirds, and two-thirds of the surface was covered with red nail polish (Estreia-Colorama, Rio de Janeiro, RJ, Brazil) to create two sound areas. All lateral areas were also covered with red nail polish, which is needed for the transverse microradiography (TMR) analysis. Subsequently, the specimens were sterilized by exposure to ethylene oxide and randomly distributed into three groups and two subgroups (n=15) according to their mean Ra values (Enamel Ra: 0.140 ± 0.029 µm and dentin Ra: 0.288 ± 0.056 µm). Specimens with Ra values below 0.100 µm or above 0.300 µm for enamel and below 0.200 µm and above 0.400 µm for dentin were excluded from the study. This was done to provide similar mean baseline Ra values among the groups.

### Study conditions and sample size calculation

The experimental models differed regarding the atmosphere, namely, microaerophilia (greenhouse with 5% Carbonic Dioxide – CO_2_), anaerobiosis (greenhouse with jar and Microbiology Anaerocult A [Merck, Darmstadt, Germany] < 0.5% O_2_), and mixed (two days in the microaerophilic model and three days in the anaerobic model). Under each atmospheric condition, half of the specimens were treated with Chlorhexidine (PerioGard - 0.12% CHX), and the other half were treated with Phosphate Buffered Saline (PBS). The entire experiment was performed in biological triplicates (n=5 each), with a final n=15 (Table 1). The sample size was calculated using the website http://powerandsamplesize.com, based on a previous study that indicated an integrated mineral loss (∆Z) of 3237.1 vol%. µm (SD: 781.0 µm) for CHX (PerioGard) versus ∆Z 6151.3 vol%. µm (SD: 1084.5) for the PBS group,^18^ under a power of 80% and α error of 5% (the size calculation was n=2).

**Table 1:** The tested atmospheres conditions and treatments in this experiment

	Atmospheres conditions	
Microaerophilia	Greenhouse with 5% CO_2_	
Anaerobiosis	Greenhouse with jar and Microbiology Anaerocult A (<0.5% O_2_)	
Mixed	2 days in the microaerophilia and 3 days anaerobiosis	
**Subgroups**	**Company/City-Country**	**Composition/Concentration**
PerioGard (positive control)	Colgate-Palmolive/São Paulo-Brazil	Active component: 0.12% chlorhexidine gluconate and sorbitol.
PBS (negative control)	-	-

The groups were divided in subgroups (n = 15).

### Preparation of artificial saliva and microcosm biofilm formation

McBain artificial saliva was prepared according to study by Braga et al.^11^ (2021). In a 24-wells microtiter plate, each enamel or dentin specimen was exposed to 1.5 mL of inoculum (human saliva-glycerol + McBain saliva, 1:50) for 8 h. After the first 8 h, the inoculum was removed, the specimens washed with PBS (5 s) and then exposed to 1.5 mL fresh medium (McBain artificial saliva) with 0.2% sucrose for 16 h, in the first 24 h. From the second to the fifth day, McBain saliva with 0.2% sucrose was replaced once a day. Before medium replacement, half of the specimens were treated daily with 0.12% CHX/PerioGard (positive control) and the other half with PBS (negative control) for 1 min (v=1 mL/well). The experiment was performed at 37 °C and each plate was stored under different atmospheric conditions.

### Colony-forming units (CFU) counting

Biofilm was washed once with PBS to remove unattached or dead bacteria. Subsequently, the biofilm was removed from the surface in microtubes containing 1 mL of 0.89% NaCl solution by sonication (Sonifier Cell Disruptor B-30, Branson, Danbury, USA) for 30 s at 20 W. For CFU counting, 100 μL of the microbial suspension were diluted to 10^-5^ and spread on petri dishes (25 µL/dish) containing three types of agar: 1) Brain Heart Infusion (BHI, Kasvi, Curitiba, Brazil) for total microorganisms; 2) Man, Rogosa, and Sharpe (MRS – Kasvi, Curitiba, Brazil) supplemented with 0.13% glacial acetic acid for total lactobacilli;^26^ and 3) SB-20M for mutans streptococci (*S. mutans* and *S. sobrinus*).^27,28^

The plates were then incubated under the same conditions as the microcosm biofilm. The plates from mixed atmosphere group were incubated separately using two atmospheres, half under anaerobiosis conditions and the other half of plates under microaerophilic conditions. After 48 h, CFU was computed and transformed to log_10_ CFU/mL.

### Transverse microradiography (TMR)

All tooth specimens were transversally sectioned and polished to obtain slices of 80–100 µm (enamel) and 100–120 µm (dentin) thickness.^21^ The slices were fixed together with an aluminum calibration step wedge. Ethylene glycol (Sigma-Aldrich, Steinheim, Germany) was applied on the dentin specimens for 24 h to avoid shrinkage.^29^ A microradiograph was obtained using an X-ray generator (Softex, Tokyo, Japan) on a glass plate at 20 kV and 20 mA (at a distance of 42 cm) for 13 min. The glass plates (high-precision photo plate; Konica Minolta Inc., Tokyo, Japan) were processed and analyzed using a transmitted light microscope fitted with a 20x objective (Zeiss, Oberkochen, Germany), a CCD (Charge-Coupled Device camera; Canon, Tokyo, Japan), and a computer. Two images per specimen were acquired using data acquisition (version 2012) and interpreted using the Inspektor Research System BV calculation software (version 2006) (Amsterdam, Netherlands). The mineral content was calculated based on the assumption of 87% volume of mineral content for sound enamel and 50% mineral content for sound dentin. Lesion depth (LD, µm) the integrated mineral loss (∆Z, vol. µm), and the average mineral loss over the lesion depth (R, vol%) were obtained.

### Statistical analysis

Data were statistically compared using the GraphPad Prism software (San Diego, CA, USA). Distribution and homogeneity were tested using the Kolmogorov–Smirnov and Bartlett’s tests, respectively. A two-way ANOVA (factors: atmospheres and treatments), was applied, followed by Tukey’s or Sidak’s test. The level of significance was set at 5%.

## Results

### CFU counting

#### Enamel

Table 2 presents the means for the CFU (log_10_/mL) for the total microorganisms, *Lactobacillus* spp., and mutans streptococci from the microcosm biofilm cultivated under various atmospheric growth conditions on the enamel specimens.

**Table 2:** Colony-forming units counting (log10 CFU/mL) of total microorganisms, *Lactobacillus* spp. and *mutans streptococci* from microcosm biofilm produced on enamel specimens

Groups	Subgroups	Total microorganisms	*Lactobacillus* spp.	*mutans streptococci*
Microaerophilia	CHX	6.38 ± 0.52^Aa^	5.87 ± 0.86^Aab^	6.41 ± 0.36^Aa^
	PBS	7.37 ± 0.43^Ba^	7.08 ± 0.11^Bab^	8.05 ± 0.12^Ba^
Anaerobiosis	CHX	7.35 ± 0.47^Ab^	6.22 ± 0.39^Abc^	6.53 ± 0.33^Aab^
	PBS	7.68 ± 0.71^Aab^	7.46 ± 0.21^Bbc^	7.35 ± 0.43^Bb^
Mixed-Anaerobic	CHX	7.15 ± 0.59^Ab^	5.69 ± 0.47^Aa^	6.88 ± 0.54^Ab^
	PBS	7.91 ± 0.25^Bb^	7.17 ± 0.10^Ba^	7.74 ± 0.11^Bab^
Mixed-Microaerophilia	CHX	7.20 ± 0.57^Ab^	6.31 ± 0.32^Ac^	6.50 ± 0.81^Aab^
	PBS	7.84 ± 0.18^Bb^	7.70 ± 0.07^Bc^	7.72 ± 0.09^Bab^

Capital letters show significant difference between treatments, for each type of atmosphere (Example: comparation between rows 3 and 4). Lowercase letters show significant difference between atmospheres, for each type of treatment (Example: comparation of between CHX rows 2, 4, 6 and 8). For total microorganisms, two-way ANOVA was applied followed by Tukey's test (treatment p<0.0001, atmosphere p<0.0001 and interaction p=0.0481). For *Lactobacillus* spp., two-way ANOVA was applied, followed by the Sidak’s test (treatment p<0.0001, atmosphere p<0.0001, no interaction, p=0.7677). For *mutans steptococci*, two-way ANOVA was applied, followed by the Tukey’s test (treatment p<0.0001, atmosphere p=0.0041 and interaction p=0.0039).

For the total microorganisms, all atmospheres, except the anaerobic condition, were able to differentiate CHX from PBS (*p*<0.0001), displaying the antimicrobial effect of the CHX. A minor difference in the growth of total microorganisms was seen in microaerophilia compared to the other atmospheres (*p*<0.0001), which did not differ from each other (anaerobic and mixed) regardless of the treatment.

Regarding the *Lactobacillus* spp. and mutans streptococci CFU*,* all atmospheres were able to differentiate CHX from PBS (*p*<0.0001), indicating the antimicrobial effect of CHX.

Lower growth of *Lactobacillus* spp. was observed under a mixed atmosphere (anaerobic CFU) than in the other conditions (*p*<0.0001), except for the microaerophilic condition. The microaerophilic atmosphere was similar to the anaerobic atmosphere but displayed lower *Lactobacillus* spp. growth when compared with the mixed atmosphere (microaerophilic CFU), which did not differ from the anaerobic and mixed-anaerobic CFU.

Regarding mutans streptococci, lower microbial growth was observed in the microaerophilic atmosphere (*p*=0.0041), which was similar to the mixed atmosphere (microaerophilic CFU). However, the microaerophilic atmosphere significantly differed from the anaerobic atmosphere when considering PBS and from the mixed atmosphere (anaerobic CFU) when considering CHX.

#### Dentin

Table 3 displays the mean for the CFU (log_10_/mL) for the total microorganisms, *Lactobacillus* spp., and mutans streptococci from a microcosm biofilm cultivated under different atmospheric growth conditions on dentin specimens.

**Table 3:** Colony-forming unit counting (log10 CFU mL) of total microorganisms, *Lactobacillus* spp. and *mutans streptococci* from microcosm biofilm produced on dentin specimens

Groups	Subgroups	Total microorganisms	*Lactobacillus* spp.	*mutans streptococci*
Microaerophilia	CHX	7.62 ± 0.22^Aa^	6.95 ± 0.46^Aab^	7.12 ± 0.39^Aa^
	PBS	7.74 ± 0.11^Aa^	7.06 ± 0.38^Aab^	7.63 ± 0.19^Ba^
Anaerobiosis	CHX	7.45 ± 0.38^Aa^	7.13 ± 0.46^Ab^	7.24± 0.35^Aa^
	PBS	7.94 ± 0.22^Ba^	7.51 ± 0.20^Ab^	7.60 ± 0.19^Ba^
Mixed-Anaerobic	CHX	7.21 ± 0.53^Aa^	7.25 ± 0.59^Ab^	7.05 ± 0.35^Aa^
	PBS	7.84 ± 0.16^Ba^	7.33 ± 0.51^Ab^	7.69 ± 0.21^Ba^
Mixed-Microaerophilia	CHX	7.49 ± 0.28^Aa^	6.76 ± 0.36^Aa^	7.21 ± 0.41^Aa^
	PBS	7.80 ± 0.16^Ba^	6.87 ± 0.56^Aa^	7.77 ± 0.22^Ba^

Capital letters show significant difference between treatments, for each type of atmosphere (Example: comparation between rows 3 and 4). Lowercase letters show significant difference between atmospheres, for each type of treatment (Example: comparation of between CHX rows 2, 4, 6 and 8). For total microorganisms, two-way ANOVA was applied, followed by Tukey's test (treatment p<0.0001, atmosphere p<0.1222 and interaction p=0.0099). For *Lactobacillus* spp., two-way ANOVA was applied, followed by the Tukey’s test (treatment p=0.0602, atmosphere p=0.0002, no interaction, p=0.5955). For *mutans streptococci*, two-way ANOVA was applied, followed by the Tukey’s test (treatment p<0.0001, atmosphere p=0.4706 and interaction p=0.4637).

For the total microorganisms CFU, all atmospheres, except microaerophilia, were able to differentiate between CHX and PBS (*p*<0.0001), showing the antimicrobial effect of CHX. When the atmospheres were compared, no differences were observed regardless of the treatment (*p*<0.1222).

Regarding *Lactobacillus* spp. CFU, none of the atmospheres was able to differentiate the effect of CHX and PBS *(p*=0.0602). When comparing the atmospheres, the mixed atmosphere (microaerophilic CFU) presented lower growth of *Lactobacillus* spp. than the other atmospheres (*p*=0.0002), except for microaerophilia.

In contrast, all atmospheres significantly showed differences between CHX and PBS on mutans streptococci growth (*p*<0.0001), whereas no differences were observed when comparing the atmospheres regardless of the treatment (*p*=0.4706).

## TMR analysis

**Figure 1:**
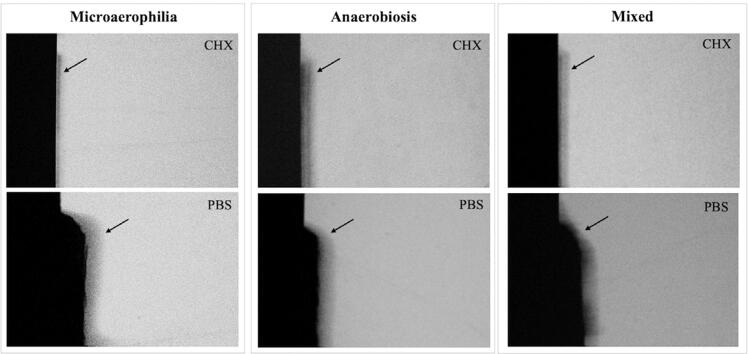
Transverse microradiography representative pictures (20×) of an enamel specimen from each group: Microaerophilia; Anaerobiosis; Mixed, after treatment with Chlorhexidine (CHX) or PBS. The arrows show the lesion area. Specimens belonging to PBS showed cavitation, regardless of the atmosphere. Specimens treated with CHX showed shallow lesion

**Figure 2:**
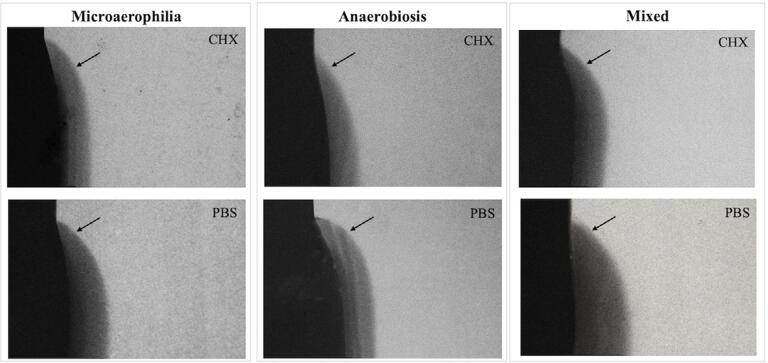
Transverse microradiography representative pictures (20×) of a dentin specimen from each group: Microaerophilia; Anaerobiosis; Mixed, after treatment with Chlorhexidine (CHX) or PBS. The arrows show the lesion area. Mixed-PBS was the only one not showing some cavitation of the dentin lesion

Tables 4 and 5 present the TMR data for the enamel and dentin specimens, respectively. Figures 1 and 2 display representative TMR images of the enamel and dentin from each group, respectively.

**Table 4:** Mean ± SD of the integrated mineral loss (ΔZ, vol%. μm), lesion depth (LD, μm) and average mineral loss (R, vol%) of the enamel specimens

Groups	Subgroups	DZ	LD	R
		**(vol%.**m**m)**	**(**m**m)**	**(vol%)**
Microaerophilia	CHX	1035.62 ± 561.38^Aa^	37.89 ± 15.46^Aa^	27.92 ± 8.16^Aa^
	PBS	7223.33 ± 1206.52^Ba^	119.84 ± 20.27^Ba^	59.14 ± 3.11^Ba^
Anaerobiosis	CHX	1702.22 ± 1056.60^Aa^	61.19 ± 24.22^Ab^	30.57 ± 10.52^Aa^
	PBS	6555.00 ± 1681.07^Ba^	134.22 ± 28.45^Bb^	54.71 ± 4.33^Ba^
Mixed	CHX	1436.11 ± 495.77^Aa^	49.46 ± 13.01^Aab^	23.94 ± 2.05^Aa^
	PBS	7246.11 ± 350.14^Ba^	119.54 ± 8.47^Bab^	59.66 ±2.06^Ba^

Capital letters show significant difference between treatments, for each type of atmosphere (Example: comparation between rows 3 and 4). Lowercase letters show significant difference between atmospheres, for each type of treatment (Example: comparation of between CHX rows 2, 4, 6 and 8). For DZ, two-way ANOVA was applied, followed by Tukey’s test (treatment p<0.0001; atmosphere p=0.7627 and interaction p=0.0168). For lesion depth, two-way ANOVA was applied, followed by Tukey’s test (treatment p<0.0001, atmosphere p=0.0148 and interaction p=0.6412). For the average mineral loss, two-way ANOVA was applied, followed by Sidak’s test (treatment p<0.0001, atmosphere p=0.7709, interaction, p=0.0284).

**Table 5:** Mean ± SD of the integrated mineral loss (ΔZ, vol%. μm), lesion depth (LD, μm) and average mineral loss (R, vol%) of the dentin specimens

Groups	Subgroups	DZ	LD	R
		**(vol%.mm)**	**(mm)**	**(vol%)**
Microaerophilia	CHX	4389.2 ± 603.5^Aa^	159.2 ± 13.7^Aa^	29.2 ± 3.4^Aa^
	PBS	5768.5 ± 523.95^Ba^	184.2 ± 24.5^Ba^	31.5 ± 2.9^Aa^
Anaerobiosis	CHX	4242.3 ± 581.68^Aa^	144.0 ± 14.0^Aa^	27.8 ±2.2^Ab^
	PBS	5149.6 ± 551.40^Bb^	194.3 ± 28.7^Ba^	28.7 ± 3.1^Ab^
Mixed	CHX	4744.3 ± 472.69^Aa^	160.3 ± 18.4^Aa^	29.0 ± 2.3^Aa^
	PBS	5692.5 ± 243.04^Ba^	183.1 ± 13.2^Ba^	31.6 ± 2.5^Ba^

Capital letters show significant difference between treatments, for each type of atmosphere (Example: comparation between rows 3 and 4). Lowercase letters show significant difference between atmospheres, for each type of treatment (Example: comparation of between CHX rows 2, 4, 6 and 8). For DZ, two-way ANOVA was applied, followed by Tukey’s test (treatment p<0.0001; atmosphere p=0.0034 and interaction p=0.2346). For lesion depth, two-way ANOVA was applied, followed by Tukey’s test (treatment p<0.0001, atmosphere p=0.809 and interaction p=0.0614). For average mineral loss, two-way ANOVA was applied followed by Tukey’s test (treatment p=0.0039, atmosphere p=0.0093, interaction, p=0.4243).

Chlorhexidine reduced enamel demineralization in all the tested atmospheres (*p*<0.0001). Similar carious lesions were produced in enamel in all atmospheres (*p*=0.7627), except for microaerophilia, which induced a shallow lesion compared to the anaerobic atmosphere, both similar to the mixed atmosphere (Table 4 and Figure 1).

Chlorhexidine was also able to reduce dentin demineralization in all tested atmospheres (*p < 0.0001*), except the average mineral loss in the case of microaerophilia and anaerobiosis. Microaerophilic and mixed atmospheres produced similar carious lesions in dentin with greater mineral loss (integrated and mean) than those produced under anaerobiosis (lesion depth *p* = 0.0809; average mineral loss *p* = 0.0093) (Table 5 and Figure 2).

## Discussion

Although the microcosm biofilm model is a well-established practice, there are several differences between the protocols, as for example the growth atmosphere. Accordingly, the atmosphere that better represents oral conditions needs to be investigated. This study did not aim to validate a type of atmosphere based on the oral environment since no comparison was made between the tested protocol and *in vivo* conditions. This study compared three experimental cultivation models (microaerophile vs. anaerobiosis vs. experimental mixed) on the colony-forming units (CFU) of the cariogenic microorganisms and tooth demineralization.

Some minor differences were observed among the atmospheric conditions, but they were able to produce very similar carious lesions in both enamel and dentin. Furthermore, most atmospheres were able to differentiate CHX vs. PBS, which is an essential outcome since the antimicrobial effect of CHX has been well established.^30^

From a microbiological perspective, we selected CFU counting – a well-established, reproducible, and simple quantitative method^31^– to analyze the effect of the atmosphere on the microorganisms from the microcosm biofilm. This method, however, is not as precise and sensitive as the Polymerase Chain Reaction (PCR) method and does not provide an overview of the entire microbiota as microbiome.^32^ Based on the findings of the CFU counting, a lower number of *Lactobacillus* spp*.* and mutans streptococci was observed under the microaerophilic than in the anaerobic environment, especially for enamel. The differences of 0.2 log_10_ CFU/mL might be clinically irrelevant; in fact, they were not significant when TMR data were taken into account.

Both bacteria were chosen because they are highly established cariogenic species.^33^ A limitation of the study is that other species, which might be present in the microcosm biofilm, were not examined. Therefore, future studies using “omics” are desirable, especially to understand the reason that CHX did not reduce *Lactobacillus* spp. in microcosm biofilms produced on dentin. Interestingly, the number of total microorganisms was reduced under anaerobic conditions for CHX. This led to speculations regarding the contribution of other species. Herein, it is important to consider that the term total microorganism does not include species that require supplementation for their growth on BHI agar.

The association between lactobacilli and dental caries dates back to one century ago.^34^ In another study, 0.2% Chlorhexidine, used in an *in situ* model, was not able to reduce the CFU counting for lactobacilli on dentin specimens when compared with the control.^35^ The author suggested that dentin can act as a “shelter” for this bacterium against the action of CHX.^35^

The outside atmosphere had little impact on the microbiological analysis of the microcosm biofilms because the biofilm itself can create its own atmosphere, with the deeper layers rich in strictly anaerobic microorganisms and the superficial layers rich in facultative microorganisms.^36^ Although dental biofilms are composed primarily of obligate anaerobe species with preferential growth in the presence of Carbon Dioxide (CO_2_), these microorganisms may be protected from the toxic effects of oxygen, enabling their growth under microaerophilic conditions.^10^ Notably, the findings of this study cannot be extrapolated to monospecies or other multispecies models.

Despite knowing the microbiological composition of the biofilm, it was clear by the tooth lesion analysis that even in the presence of different species, the set of metabolic products released into the biofilms under different atmospheres was able to create similar artificial carious lesions. Metabolome analysis could be used to better discuss the results; however, the most important response variable is related to the tooth (TMR data).

Regarding enamel demineralization, the mineral loss (integrated and mean) was similar among different atmospheres; however, the anaerobic condition induced a deeper lesion, showing that, in this case, the acids could have penetrated deeper into the environment, which may be related to the metabolic profile of the biofilm. While dentin lesion depth was similar among the atmospheres, dentin mineral loss (integrated and mean) was lower for anaerobic than for microaerophilic and mixed atmospheres, which behaved similarly. Therefore, as previously discussed, the metabolic products (primarily acids) induced by the anaerobic biofilm may have been neutralized by the organic content of the dentin.^3^

The mixed biofilm model was first performed by Braga, et al.^11^ (2021) but in a different sequence: the first three days in an anaerobic and the last two days in a microaerophilic atmosphere. In their study, the biofilm model was able to differentiate CHX from PBS. The results of this study warrant further metabolome analysis to better explain the slight differences found in CFU counting and TMR analysis and to signalize that the mixed model can be applied in the future.

For both tissues, especially the enamel, lesions induced in the absence of treatment (PBS group) were highly demineralized, and most of them were cavitated (about 85–100% for enamel and 31–55% for dentin), with no differences among the atmospheres. It is suggested that the higher cavitation of enamel is due to its lower organic content compared to dentin, which plays an important role in the modulation of de-remineralization progression. Also, the percentage of prevention fraction of CHX was much higher for enamel (~78%) than for dentin (~22%), which may be related to the high degree of de-remineralization of the enamel and the type of interaction of CHX with the biofilm, in agreement with previous reports.^16,18^

Our results provide new information about the effect of the atmosphere on microcosm biofilm growth and its potential to induce tooth demineralization. These different protocols should be analyzed in the future to identify their impact on the results and interpretation of metabolome. Moreover, the biofilm should be analyzed by laser scanning microscopy or scanning electron microscopy to map the 3D architecture of the biofilm and thus identify if the specific organization of microbial communities could be created by different atmospheric conditions.

## Conclusion

In conclusion, this study identified some minor differences among the atmospheric conditions; however, in general, microcosm biofilms produced under microaerophilia, anaerobiosis, or mixed models produced very similar artificial carious lesions in both enamel and dentin. Considering a general overview, mixed model may be interesting since it does not differ from the classical ones; however, researchers must be prepared to combine both methods in the Laboratory. Nevertheless, any of them could be applied since all were able to produce dental caries and to differentiate CHX and PBS.
